# Adsorption of acrolein, propanal, and allyl alcohol on Pd(111): a combined infrared reflection–absorption spectroscopy and temperature programmed desorption study

**DOI:** 10.1039/c6cp00877a

**Published:** 2016-05-06

**Authors:** Karl-Heinz Dostert, Casey P. O'Brien, Francesca Mirabella, Francisco Ivars-Barceló, Swetlana Schauermann

**Affiliations:** a Fritz-Haber-Institut der Max-Planck-Gesellschaft , Faradayweg 4-6 , 14195 Berlin , Germany . Email: schauermann@fhi-berlin.mpg.de; b Institut für Physikalische Chemie , Christian-Albrechts-Universität zu Kiel , Max-Eyth-Str. 2 , 24118 Kiel , Germany

## Abstract

Atomistic-level understanding of the interaction of α,β-unsaturated aldehydes and their derivatives with late transition metals is of fundamental importance for the rational design of new catalytic materials with the desired selectivity towards C=C *vs.* C=O bond partial hydrogenation. In this study, we investigate the interaction of acrolein, and its partial hydrogenation products propanal and allyl alcohol, with Pd(111) as a prototypical system. A combination of infrared reflection–absorption spectroscopy (IRAS) and temperature programmed desorption (TPD) experiments was applied under well-defined ultrahigh vacuum (UHV) conditions to obtain detailed information on the adsorption geometries of acrolein, propanal, and allyl alcohol as a function of coverage. We compare the IR spectra obtained for multilayer coverages, reflecting the molecular structure of unperturbed molecules, with the spectra acquired for sub-monolayer coverages, at which the chemical bonds of the molecules are strongly distorted. Coverage-dependent IR spectra of acrolein on Pd(111) point to the strong changes in the adsorption geometry with increasing acrolein coverage. Acrolein adsorbs with the C=C and C=O bonds lying parallel to the surface in the low coverage regime and changes its geometry to a more upright orientation with increasing coverage. TPD studies indicate decomposition of the species adsorbed in the sub-monolayer regime upon heating. Similar strong coverage dependence of the IR spectra were found for propanal and allyl alcohol. For all investigated molecules a detailed assignment of vibrational bands is reported.

## Abstract 2

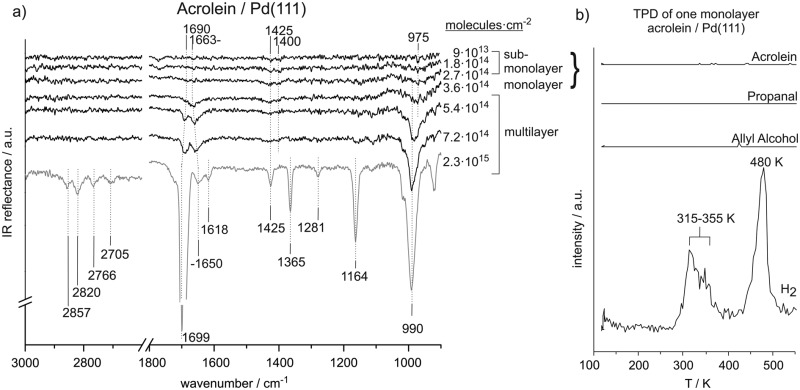
We present a mechanistic study on adsorption of acrolein and its partial hydrogenation products propanal and allyl alcohol over Pd(111) to understand the factors governing the selectivity in acrolein hydrogenation.

## 1. Introduction

The atomistic-level understanding of factors governing the selectivity in partial selective hydrogenation of α,β-unsaturated aldehydes and ketones over transition metal surfaces is of fundamental importance for numerous industrial processes.^1^ The primary hydrogenation products of this class of reactions are either a saturated aldehyde or an unsaturated alcohol. To avoid the formation of undesired products (mostly saturated aldehydes and ketones) and thereby an often difficult and cost-intensive separation process, a high selectivity in C=C or C=O bond hydrogenation is desirable.

It is widely accepted that the adsorption geometry of the reactant on the catalyst surface is an important factor governing the selectivity of the hydrogenation reaction.^2–7^ On the one hand, the adsorption geometry of an α,β-unsaturated aldehyde or ketone can be manipulated in favor of C=O bond hydrogenation by adding bulk substituents to the C=C functional group.^1,2,8^ Acrolein, however, is the most difficult α,β-unsaturated carbonyl compound to selectively hydrogenate the C=O bond because the C=C group is not sterically hindered by any substituent. On the other hand, the structure of the catalyst also influences the adsorption geometry of the reactant and thus may have a decisive influence on the selectivity of the conversion. Despite this fact, the effect of the catalyst structure on the selectivity in partial hydrogenation is much less understood than the effects of the molecular structure.

Our recent work on acrolein hydrogenation over a Pd(111) surface under well-defined ultrahigh vacuum (UHV) conditions provided new insight into the relationship between the catalyst structure and its selectivity in partial hydrogenation of acrolein. For the first time we showed that a near 100% selectivity towards hydrogenation of C=O in acrolein is possible over Pd(111), while no unsaturated alcohol was formed over Pd nanoparticles.^9^ This was a particularly unexpected result, since numerous studies on powdered Pd catalysts have previously shown near 100% selectivity towards C=C bond hydrogenation in α,β-unsaturated carbonyl compounds.^10,11^ We explain the unexpected selectivity of Pd(111) by formation of a dense layer of a spectator species – oxopropyl groups – that is formed during the initial stages of the reaction and turns the surface highly selective toward C=O bond hydrogenation.

Infrared spectroscopy is a powerful method for investigating the adsorption geometry of reactants, and their products, on well-defined catalyst surfaces. Vibrational modes of acrolein, propanal, allyl alcohol, and molecules with similar functional groups were assigned in a number of previous studies. A general overview of typical vibration frequencies can be found in a textbook by Colthup, Daly, and Wiberley.^12^ Hamada *et al.* assigned IR absorption features of gas-phase acrolein to calculated vibration frequencies in the range from 4000 cm^–1^ to 400 cm^–1^.^13^ Puzzarini *et al.* studied the gas-phase structures of acrolein and their IR vibration frequencies theoretically and compared them to experimental results. Several IR absorption features were found to significantly change from *trans*- to *cis*-acrolein.^14^ However, Puzzari assigned IR absorption features of vinyl- and aldehyde-C–H bend modes in a way opposite to Hamdada,^13^ Fujii,^15^ and Akita.^16^ Fujii *et al.* ^15^ and Akita *et al.* ^16^ investigated the structures of acrolein after adsorption on silver and gold films under UHV conditions. In both studies, IR vibrational modes were assigned in the range from 1800 cm^–1^ to about 800 cm^–1^. Particularly, a strong intensity of the CH_2_ wag vibration peak for adsorbates with molecular planes parallel to the metal surface was observed. Osaka *et al.* investigated the adsorption of 1,3-butadiene on Au(111) and Ag(111) surfaces.^17^ Loffreda *et al.* studied the adsorption of acrolein on Pt(111) by total energy and frequency calculations combined with high resolution electron energy loss spectroscopy (HREELS) experiments.^18^ C–H stretching modes of surface species during hydrogenation of acrolein and other α,β-unsaturated aldehydes were studied by sum-frequency generation spectroscopy by Kliewer *et al.* ^19^

Thorough experimental and theoretical studies on the vibrational modes of propanal were published by Guirgis *et al.* ^20^ and Frankiss *et al.* ^21^ Guirgis studied the conformational stability and assigned vibrational modes in liquid xenon at wavenumbers up to 3500 cm^–1^ and Frankiss investigated the vibrational modes of normal and deuterium-labeled propanal in the gas phase. In an earlier study, vibrations of liquid and crystalline propanal were assigned experimentally by Sbrana.^22^ In a more general investigation, Byrne *et al.* studied carbonyl vibration frequencies and C–H vibrations of saturated aliphatic aldehydes.^23^ Guirgis, Frankiss, Sbrana, and Byrne pointed out two typical features in the C–H stretching region, which result from Fermi resonance between the CH bend overtone and the CH stretching fundamental at 2700–2770 cm^–1^ and 2800–2870 cm^–1^. This characteristic IR absorption of the aldehyde-CH group was studied in more detail by Pinchas^24^ and Eggers *et al.* ^25^

A very detailed assignment of the vibrational modes of normal and isotopically labeled allyl alcohol in the gaseous, liquid, and glassy states as well as in argon and nitrogen matrices in the range from 4000 cm^–1^ to 200 cm^–1^ was performed by Silvie and Perchard. In this study, *cis* and *gauche* conformers have been identified by their IR vibration frequencies.^26^ In a more recent study, Durig *et al.* assigned vibrational frequencies from theoretical calculations as well as from IR and Raman experiments for the four allyl alcohol conformers *gauche–trans*, *gauche–gauche*, *cis–trans*, and *cis–gauche*.^27^

Despite these previous combined experimental and theoretical efforts, there is still a lack of understanding of how acrolein and its derivatives change their structure upon interaction with transition metal surfaces and how their adsorption geometry depends on the adsorbate coverage. In this report, we present a comprehensive study on adsorption of acrolein, propanal, and allyl alcohol on a Pd(111) single crystal. Infrared reflection–absorption spectroscopy (IRAS) has been employed to investigate the molecular structures and adsorption geometries of the adsorbates as a function of adsorbate coverage. The particular focus of this study lied on determining the degree of molecular bonds perturbation upon interaction of the gas phase molecules with Pd that might have a decisive influence on the further chemical transformations of the surface species. The desorption and decomposition of the adsorbates on Pd(111) has been monitored by temperature programmed desorption (TPD) experiments.

## 2. Experimental details

All experiments have been performed at the Fritz-Haber-Institut, Berlin, in a UHV apparatus that was described in detail before.^28^ In brief, acrolein, propanal, and allyl alcohol have been dosed onto the sample through a doubly differentially pumped multi-channel array source controlled by valves and shutters. The source has been operated at room temperature, and the beam diameter has been chosen to exceed the sample size. The Pd(111) single crystal has been cleaned prior to use by repeated cycles of Ar^+^ ion bombardment at room temperature, annealing at 1000 K and oxidation in 1 × 10^–6^ mbar O_2_ at 750 K to remove residual carbon. The final cleaning cycle has been stopped after annealing. The flatness and cleanliness of the Pd(111) single crystal surface has been checked by low energy electron diffraction (LEED) and IRAS measurements of adsorbed CO.

IRAS data have been acquired using a vacuum Fourier-Transform infrared (FT-IR) spectrometer (Bruker IFS 66v/S) with a spectral resolution of 2 cm^–1^ and a mid-infrared (MIR) polarizer and p-polarized IR light. TPD experiments have been carried out in the same vacuum system and by using an automated quadrupole mass spectrometer (QMS) system (Hiden Analytics) to monitor the partial pressures of the desorbing molecules: acrolein (parent ion at 56 a.m.u.), H_2_: (2 a.m.u.), propanal (parent ion and main fragment at 58 a.m.u.), propenol (parent ion at 58 a.m.u., main fragment at 57 a.m.u. and further prominent fragment at 31 a.m.u.), propanol (parent ion at 60 a.m.u., main fragment at 31 a.m.u.). Prior to each TPD cycle, a blank experiment was carried out to monitor feasible evolution of hydrogen from the heating element and other sources. Some minor hydrogen evolution was obtained at the very beginning of the heating cycle (close to 100 K), which results in minor hydrogen signals (2 amu) that can be seen in all TPD data. These small peaks have to be considered as an experimental artefact. No hydrogen evolution at temperatures higher that 200 K was obtained in the blank experiments.

Shortly before each experiment the sample has been flash annealed to 600 K before cooling to 120 K. Acrolein (Fluka, 95% purity), propanal (Acros Organics, >99% purity), and allyl alcohol (Aldrich, >99% purity) have been purified prior to their exposure by repeated freeze–pump–thaw cycles.

## 3. Results

In this study, the adsorption of acrolein, propanal, and allyl alcohol was investigated on Pd(111) at 120 K under well-defined UHV conditions by IRAS. The molecular structures and the adsorption geometry of the adsorbates were studied as a detailed function of coverage. To obtain a reference for an unperturbed molecule, the IR spectra were recorded at multilayer coverages, at which the majority of the adsorbed molecules do not directly interact with the surface. These reference spectra were compared with those obtained at sub-monolayer coverages to investigate the perturbation of chemical bonds by the interaction with Pd.

It is important to note that the adsorption geometry of adsorbates on metal surfaces can be deduced from their IR spectra based on the metal surface selection rule (MSSR).^29–31^ According to the MSSR, only the component of the dynamic dipole moment perpendicular to the metal surface can be detected, while vibrations parallel to the surface are strongly attenuated by formation of an image dipole in the substrate. Hence, characteristic intensity distributions of IR absorption peaks in sub-monolayer coverage regime can give information on the adsorption geometry of the corresponding adsorbate.

Three main spectral regions can be distinguished for all investigated molecules, which are characteristic for C–H stretching vibrations (3100–2700 cm^–1^), C=O and C=C stretching vibrations (1800–1600 cm^–1^) as well as C–H deformation and C–C stretching vibrations (<1500 cm^–1^). The exact assignment of the IR vibration peaks for each compound will be discussed in the following sections.

### 3.1. Adsorption of acrolein

**Fig. 1:**
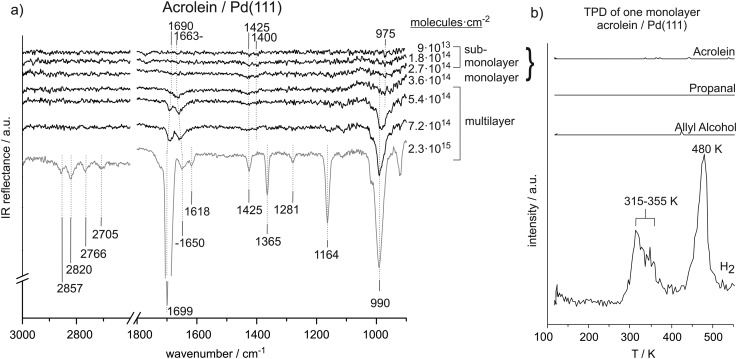
(a) IR spectra of acrolein on Pd(111) from sub-monolayer to multilayer coverage recorded at 120 K. (b) TPD study after adsorption of a monolayer of acrolein (3.6 × 10^14^ cm^–2^) on Pd(111).

Fig. 1(a) shows the coverage-dependent evolution of IR spectra of acrolein on Pd(111) from the sub-monolayer to the multilayer regime. The numbers next to the spectra indicate the particular acrolein coverages expressed as an absolute number of adsorbed acrolein molecules per cm^2^, which was determined based on sticking coefficient measurements and the absolute molecular beam flux. Table 1 summarizes the assignment of vibrational modes to the observed IR absorption features of the different acrolein species on the Pd(111). In this table, we tentatively distinguish four different species, which we refer to as types A, B, C, and D. Coverages up to 2.7 × 10^14^ molecules per cm^2^ (corresponding to less than 0.2 acrolein molecules per surface Pd atom; species A and B in Table 1), at which the most strongly perturbed species appear, can be assigned to the sub-monolayer regime. The surface coverage of 3.6 × 10^14^ molecules per cm^2^ (corresponding to 0.25 acrolein per surface Pd atom; species C in Table 1) is related to the full monolayer coverage. Finally, the multilayer adsorption regime (species D in Table 1) starts from the exposure of 5.4 × 10^14^ molecules per cm^2^. With increasing exposure, constantly growing intensities of all vibrational features were observed for species D that confirm the formation of acrolein ice. The IR spectra of species D can serve as a reference for an unperturbed molecule.

**Table 1:** Assignment of IR vibrational modes of acrolein on Pd(111) at 120 K

Mode	IR frequency/cm^–1^				Ref./cm^–1^
	Species A (sub-ML)	Species B (sub-ML)	Species C (∼1 ML)	Species D (multilayer)	
*ν*(CH)_v_					3069,^13^ 3092–3077^12^
*ν*_a_(CH_2_)				2857	3103,^13^ 3092–3077^12^
*ν*_s_(CH_2_)				2820	2998,^13^ 3025–3012^12^
2*δ*(CH)_C=O_				2766^*a*^	2772,^24^ 2800^13^
					2830–2810^12^
					2867–2818^20^
*ν*(CH)_C=O_				2705^*a*^	2718,^24^ 2716^21^
					2974–2712^20^
					2720–2695^12^
*ν*(C=O)			1663–1650	1700–1690	1672–1670 (on Ag)^16^
					1684–1670 (on Ag)^15^
					1724 (gas)^13^
*ν*(C=C)				1618	1618–1603,^15^ 1625^13^
					1644–1617^12^
*δ*(CH_2_)	1400	1425	1425	1425	1425,^16^ 1420^13,14,18^
					1431–1425^15^
*δ*(CH)_C=O_				1365	1365,^16^ 1360,^13^
					1365–1360,^15^ 1275^14^
*δ*(CH)_C=C_				1281	1281,^16^ 1284^15^
					1275,^13^ 1360^14^
*ν*(C–C)				1164	1169–1159,^15^ 1158^13,14^
					1165–1159^16^
*ω*_T_(HC=CH_2_)	(975)	(975)	(990)	(990)	1018–841^15^
					1020–950^16^
					1022–1002^17^ ^*b*^
					993,^13^ 995–985^12^

^*a*^Fermi resonance.

^*b*^Vibration in 1,3-butandiene.

#### 3.1.1. Unperturbed acrolein in multilayer

First, we will address the multilayer regime of acrolein adsorption and identify the associated vibrational bands characteristic to a nearly unperturbed acrolein molecule. Here, a large number of IR absorption modes can be distinguished. In the C–H stretching region, IR vibrations are observed at 2857 cm^–1^, 2820 cm^–1^, 2766 cm^–1^, and 2705 cm^–1^. Based on the previous studies, we assign the two higher wavenumber peaks to CH_2_ asymmetric (2857 cm^–1^) and symmetric stretching (2820 cm^–1^) modes. Previously, the two CH_2_ stretching modes were observed near 3000 cm^–1^ and 3100 cm^–1^ and thus at higher frequencies.^12,13^ The two features at 2766 cm^–1^ and 2705 cm^–1^ can be assigned to the first overtone of the aldehyde-C–H bend and the aldehyde-C–H stretching fundamental vibrations. The appearance of the two peaks is known to typically appear in aldehydes and was previously explained by strong Fermi resonance.^12,20,24^

In the C=C and C=O stretching region, a very pronounced IR vibration is observed at 1690–1700 cm^–1^ and weaker bands are observed at 1650 cm^–1^ and 1618 cm^–1^. The bands at 1690–1700 cm^–1^ and at 1650 cm^–1^ are assigned to the C=O stretching modes at multilayer and monolayer acrolein coverages, respectively. The frequency of the C=O vibration at multilayer acrolein coverages increases with coverage from 1690 cm^–1^ at 5.4 × 10^14^ molecules per cm^2^ to 1699 cm^–1^ at 2.3 × 10^15^ molecules per cm^2^ and thus shifts closer to the previously reported value of 1724 cm^–1^ for acrolein in the gas phase.^13^ The C=O vibrational band associated with a monolayer coverage of acrolein at 1650 cm^–1^ is red-shifted relative to that in multilayer acrolein due to the interaction of the C=O vibration with the surface. The C=C stretching vibration at 1618 cm^–1^ appears in the frequency range reported in literature.^13,15^ Note that the intensity of the C=C vibrational band is considerably lower than that one of the C=O bond due to significantly lower dynamic dipole moment. It should be noted that previously published experimental assignment of acrolein gas-phase modes suggest no significant coupling between C=O and C=C vibrations.^32^ It should be kept in mind, however, that this can change, especially when acrolein is adsorbed on metals, and the modes should be considered rather as coupled modes having mostly C=C or C=O character.

In the region below 1500 cm^–1^, well-separated IR absorption features appear at 1425 cm^–1^, 1365 cm^–1^, 1281 cm^–1^, 1164 cm^–1^, and 990 cm^–1^. The precise assignment of the bands in this vibrational region is quite difficult due in part to contradictory assignments of these bands in the literature. Based on the previous reports, the vibration at 1425 cm^–1^ can be assigned to the CH_2_ scissor bending mode and the IR absorption at 990 cm^–1^ to a deformation of the HC=CH_2_ unit, possibly a *trans*-wag mode, which is a =CH_2_ twist coupled with a H–C= out-of-plane bend vibration. The vibration at 1164 cm^–1^ is related to the C–C stretching vibration. The vibrational modes at 1365 cm^–1^ and 1281 cm^–1^ can be assigned to bending of the aldehyde- and vinyl-C–H bonds. Previously, most studies assigned the higher wavenumber peak to the aldehyde-C–H group and the lower frequency peak to the vinyl-C–H group;^12,13,16,18^ however, we also found the reversed assignment.^14^ In literature, the C–C stretching was observed at 1169–1158 cm^–1^ ^13–16^ and the CH_2_ scissor deformation in acrolein was reported near 1425 cm^–1^.^13,14,16^ The strong IR absorption near 975–990 cm^–1^ was related to the HC=CH_2_ unit; however, previous publications assigned these features to different modes, such as CH bend,^15,16^ CH_2_ wag^16^ or CH_2_ twist^17^ vibrations. Colthup *et al.* referred to this intense IR absorption as HC=CH_2_ *trans*-wag mode.^12^

#### 3.1.2. Acrolein at monolayer coverage

Significant changes in the infrared spectrum of acrolein adsorbed on Pd(111) occurs when the coverage is decreased from multilayers, in which the molecules are relatively unperturbed, to monolayer coverage. At monolayer coverage, the most prominent peak is observed at 1663 cm^–1^ and thus in the typical range for a C=O stretching vibration. It shifts to 1650 cm^–1^ with increasing coverage. The position of this band is about 30–40 cm^–1^ lower than the C=O stretching vibration in acrolein multilayer and about 60 cm^–1^ lower than the C=O stretching frequency determined in previous studies on acrolein in the gas phase (1724 cm^–1^).^33^ Further noticeable vibrations are the CH_2_ scissor vibration at 1425 cm^–1^ and the HC=CH_2_ *trans*-wag mode near 990 cm^–1^. These frequencies are similar to those for acrolein adsorbed in multilayers. Nearly zero intensity is observed for the bands at 1365 cm^–1^ and 1164 cm^–1^ and the C–H stretching vibrations in the range from 2705 cm^–1^ to 2857 cm^–1^.

#### 3.1.3. Acrolein in sub-monolayer coverage

In the sub-monolayer regime further pronounced changes occur as compared to unperturbed acrolein. Most prominently, no vibrational features in the range of C=O and C=C vibrations are observed. Low intensity IR vibrations are detected at 1425 cm^–1^, 1400 cm^–1^, and 975 cm^–1^. All of these IR absorption peaks can assigned to typical deformation modes of the CH_2_ group. Based on the previous reports, we assign the vibrations at 1425 cm^–1^ and 1400 cm^–1^ to CH_2_ scissor bend vibrations and the intense IR absorption at 975 cm^–1^ to a deformation of the *trans*-wag mode of the HC=CH_2_ unit.

The vibration at 1400 cm^–1^ appears at relatively low wavenumber compared to typical CH_2_ scissor frequencies reported in literature and thus points to a possible perturbation of the CH_2_ group. We refer to this species as type A in Table 1. This perturbation becomes less pronounced, and the band shifts to a frequency characteristic of an unperturbed molecule (1425 cm^–1^) with increasing coverage. We refer to this surface species with less perturbed CH_2_ group as to species B. However, it should be kept in mind that the transition from A to B arises most likely from the increasing coverage and a concomitant increase of a dipole–dipole coupling that might results in the frequency shifts. The distinguishing between the species A and B should not be misunderstood as referring to two chemically and/or structurally different adsorbates but rather to the same surface species merely exhibiting different vibrational frequency due to dipole coupling effects. The IR absorption at 975 cm^–1^ appears in all IR spectra recorded in sub-monolayer regime. Note that the first two spectra also contain a band at 1780 cm^–1^, whose intensity is comparable to the bands related to the C–H deformation vibrations (1400–1425 cm^–1^). As this band completely disappears with growing acrolein coverage and is not present in acrolein multilayer spectra, this vibration is most likely related to some surface contamination (*e.g.*, CO molecules adsorbed from the background), which is replaced by acrolein with growing exposure.

#### 3.1.4. TPD of acrolein

Fig. 1(b) shows a TPD experiment carried out on Pd(111) with pre-adsorbed of 1 ML acrolein (3.6 × 10^14^ cm^–2^ molecules cm^–2^). No desorption of acrolein, allyl alcohol or propanal were detected; however, pronounced hydrogen peaks appear at between 315 and 355 K and at 480 K, indicating acrolein decomposition. This observation points to a complete decomposition of acrolein monolayer upon heating. Note that surface adsorbed hydrogen desorbs from clean Pd(111) surface at 300 K, so that peak at 315–355 K observed in our experiment cannot be desorption limited and must arise from acrolein decomposition. This desorption pattern consisting of two peaks is typical for decomposition of a variety of hydrocarbons on Pd.^34^ Previously we investigated the temperature-programmed desorption and decomposition of isophorone on Pd(111) and observed a similar sequential decomposition resulting in H_2_ desorption peaks at 325–345 K and 485 K.^35^ Decomposition of acrolein to hydrogen does not result in self-hydrogenation of intact acrolein molecules as it was previously reported for some other hydrocarbons,^36^ as indicated by absence of allyl alcohol or propanal in the desorption traces.

### 3.2. Adsorption of propanal

**Fig. 2:**
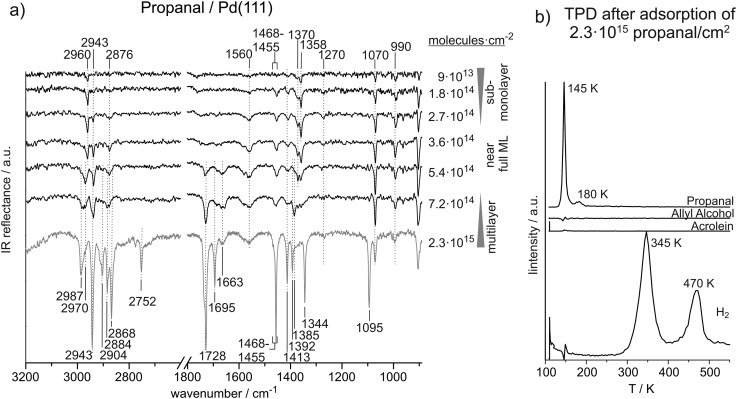
(a) IR spectra of propanal on Pd(111) from the sub-monolayer to multilayer regime recorded at 120 K. (b) TPD after deposition of about six layers of propanal on Pd(111).

Fig. 2(a) shows the coverage dependent evolution of IR spectra of propanal on Pd(111) from the sub-monolayer to the multilayer coverage at 120 K. The absolute propanal coverages are located to the right of the corresponding spectra. Coverage-dependent changes of IR absorption features suggest three coverage regimes: sub-monolayer, monolayer, and multilayer. The formation of a complete monolayer requires the propanal exposures in the range of 3.6 × 10^14^ to 5.4 × 10^14^ molecules per cm^2^; above this coverage the propanal multilayer is formed. A clear sub-monolayer regime is observed below 2.7 × 10^14^ propanal molecules per cm^2^. Table 2 gives an overview of all IR vibrational peaks at sub-monolayer, monolayer and multilayer propanal coverages observed in this study and an overview of the assignments from literature.^12,20–24,37–40^

**Table 2:** Assignment of IR vibrations of propanal on Pd(111) at 120 K

Mode	IR frequency/cm^–1^			Ref./cm^–1^
	Sub-monolayer	Near monolayer	Multilayer	
*ν*_a_(CH_3_)	2960	2970	2987	2992–2985,^20^ 2980,^21^ 2982–2980^22^
*ν*_a_(CH_2_)	2943	2943	2943	2954–2941,^20^ 2941–2939^22^
*ν*_s_(CH_2_)			2904	2914–2901,^20^ 2909–2899^22^
*ν*_s_(CH_3_)	2876		2884	2905–2883,^20^ 2880^21^
*ν*(CH)			2868^*a*^	2774–2712 + 2867–2818^20^
			2752^*a*^	2700 + 2800^21^
				2850–2700^23^
2*δ*(CH)				2718 + 2772^24^
*ν*(C=O)	1560	1663	1728/1695	1580 (Cu-acetylacetone)^37^
				1595, 1562 (carboxylate)^38^
				1569^*b*^ (acrolein/Pt-Ni)^39^
				1565 (CO_2_^–^)^40^
				1740–1720,^12^ 1754–1729^20^ 1746,^23^ 1750^21^
*δ*_a_(CH_3_)	1455–1468	1455–1468	1455–1468	1475–1450^12^
				1467–1451^20^
				1460, 1451^21^
*δ*(CH_2_)	1413	1413	1413	1425,^12^ 1423–1413^20^ 1420^21^
*δ*(CH)	1370	1370	1392	1395,^21^1381–1374,^20^ 1385,^25^ 1410–1380^12^
*δ*_s_(CH_3_)	1358	1385		1342–1338,^22^ 1383–1377,^12^ 1380^21^
				1395–1392^20^
*ω*(CH_2_)			1344	1340^21^
*τ*(CH_2_)	1270			1261–1250,^20^ 1250,^21^ 1260^22^
*ρ*(CH_3_)	1070	1070	1095	1098–1093^20^
*ν*_a_(CCC)	990			1001–993^20^

^*a*^Fermi resonance.

^*b*^In HREELS measurement.

#### 3.2.1. Unperturbed propanal molecule in multilayer

At multilayer propanal coverage, several modes are observed at frequencies typical for C–H stretching vibrations. IR absorption peaks appear at 2987 cm^–1^, 2970 cm^–1^, 2904 cm^–1^, 2884 cm^–1^, 2868 cm^–1^, and 2752 cm^–1^. IR absorption near 2987 cm^–1^, 2943 cm^–1^, 2904 cm^–1^, and 2987 cm^–1^ appear at typical frequencies for CH_3_ and CH_2_ stretching vibration modes. Based on assignments of IR vibrations of propanal reported in literature^20–22^ and on our previous combination of density functional theory (DFT) and IRAS studies on adsorbed isophorone,^41^ the vibrations at 2987 cm^–1^ and 2884 cm^–1^ are assigned to CH_3_ asymmetric and symmetric stretching, correspondingly, and the IR absorption at 2943 cm^–1^ and 2904 cm^–1^ are related to the CH_2_ asymmetric and symmetric stretching modes. The features at 2868 cm^–1^ and 2752 cm^–1^ are both assigned to the aldehyde-C–H group. As earlier discussed for acrolein, these two peaks are known to result from strong Fermi resonance between the first overtone of the CH bending and CH stretching fundamental.^20,21,23,24^ The CH_3_ asymmetric (2987 cm^–1^) and symmetric stretching (2884 cm^–1^) vibrations are in the range of those previously reported at 2992–2980 cm^–1^ ^20–22^ and 2905–2880 cm^–1^,^20,21^ respectively. The CH_2_ asymmetric stretching was found at 2914–2899 cm^–1^.^20,22^ However, none of the literature data refers to propanal on a metal surface.

In the C=O stretching region, we observe two features growing simultaneously at 1728 cm^–1^ and 1695 cm^–1^. The band at 1728 cm^–1^ is within the range (1754–1720 cm^–1^) of previously assigned^12,20,21,23^ C=O stretching vibrations of unperturbed propanal molecules, suggesting a mostly unperturbed C=O group in multilayer propanal coverages. The vibration at 1695 cm^–1^, however, indicates the simultaneous formation of a species with a slightly weakened C=O bond and thus to the formation of an adsorbate strongly interacting with the surface.

In the region below 1500 cm^–1^, IR absorption features are observed at 1455–1468 cm^–1^, 1413 cm^–1^, 1392 cm^–1^, 1285 cm^–1^, 1344 cm^–1^, and 1095 cm^–1^. The vibrations at 1455–1468 cm^–1^ and 1413 cm^–1^ are clearly related to CH_3_ asymmetric bending and CH_2_ bending modes and the feature at 1392 cm^–1^ may relate to the CH_3_ symmetric bending and/or CH bending.^12,20–22^ The vibration at 1344 cm^–1^ indicates a CH_2_ wag mode and the peak at 1095 cm^–1^ reveals a CH_3_ rock vibration. The vibrational frequencies agree well to values from literature. Previously, CH_3_ asymmetric and symmetric bending were found in the ranges from 1475–1413 cm^–1^ ^12,20,21^ and 1395–1338 cm^–1^.^20–22^ CH_2_ bending was reported at 1425–1413 cm^–1^ ^12,20,21^ and CH_2_ wagging at 1340 cm^–1^.^21^ CH bending was found at 1410–1374 cm^–1^ ^12,20,21,25^ and CH_3_ rocking was found at 1098–1093 cm^–1^.^20^

#### 3.2.2. Propanal at monolayer coverage

The number of IR vibrational peaks decreases as the propanal coverage is decreased from multilayer (2.3 × 10^15^ molecules per cm^2^) to near-multilayer coverage (3.6 × 10^14^ molecules per cm^2^). Based on the absolute number of adsorbed molecules (0.36–0.25 propanal molecules per surface Pd atom) and strong changes of the IR spectral features, we refer to this coverage regime as to the near-monolayer coverage. As compared to unperturbed propanal in a multilayer, the IR absorption features assigned to the CH_3_ and CH_2_ symmetric stretching modes disappear close to a monolayer coverage as well as the peaks related to the CH group. The absence of these vibrational modes is most likely explained by a parallel orientation of the respective dynamic dipole moments to the metal surface, which would result in the vanishing of this vibration due to the MSSR.^42^ The vibration at 2943 cm^–1^, which has been related to the CH_2_ asymmetric stretching in propanal multilayers, remains at the same frequency. The CH_3_ asymmetric stretching frequency, in contrast, decreases from 2987 cm^–1^ at multilayer coverage to 2970 cm^–1^ at monolayer coverage.

At near-monolayer coverage, the C=O stretching frequency (1663 cm^–1^) is significantly lower than that of multilayer coverages (1728 and 1695 cm^–1^), which suggests a significant weakening of the C=O bond and hence to a strong interaction of this group with the Pd surface.

In the region of the bending vibrations, peaks are observed at 1455–1468 cm^–1^, 1413 cm^–1^, 1385 cm^–1^, 1370 cm^–1^, and 1070 cm^–1^. Similar to those observed at higher coverages, the vibrations at 1455–1468 cm^–1^ and 1413 cm^–1^ are assigned to CH_3_ asymmetric bending and the CH_2_ bending. However, the CH_3_ symmetric bending and CH bending modes can tentatively be resolved as the two separated features at 1385 cm^–1^ and 1370 cm^–1^. The vibration at 1070 cm^–1^ is assigned to a CH_3_ rocking mode which is slightly shifted to lower frequencies relative to higher coverages.

#### 3.2.3. Propanal at sub-monolayer coverage

Compared to acrolein, a relatively large number of IR absorption features are observed at low propanal concentration on the Pd(111) surface. Several prominent IR vibrational peaks appear in the sub-monolayer regime and saturate near the monolayer coverage. We assume that these vibrational modes are related to adsorbates that are formed in the first monolayer and remain present on the surface at multilayer coverages.

At surface coverages ranging from 9 × 10^13^ to 2.7 × 10^14^ molecules per cm^2^ (0.06–0.18 propanal molecules per surface Pd atom), pronounced IR absorption peaks appear in the region of the C–H stretching vibrations at 2960 cm^–1^, 2943 cm^–1^, and 2876 cm^–1^. The mode at 2960 cm^–1^ is assigned to the CH_3_ asymmetric stretching and the vibration 2876 cm^–1^ is related to the CH_3_ symmetric stretching. Thus, the asymmetric stretching mode appears at 27 cm^–1^ lower frequency compared to the multilayer species while the symmetric mode only decreases by 8 cm^–1^. The frequency of the CH_2_ asymmetric stretching at 2943 cm^–1^ is found to be independent of the surface coverage.

In the region typical of C=O stretching vibrations, no IR absorption features can be identified at sub-monolayer coverages. At 1560 cm^–1^, however, an IR vibration is observed, which is present neither in propanal nor in other aldehydes and can therefore not be related to any vibration of intact propanal molecules. Nevertheless, similar vibration frequencies have been observed before. Murillo *et al.* observed a vibration at 1569 cm^–1^ in HREELS experiments of acrolein adsorbed on Pt–Ni–Pt(111) and Pt–Co–Pt(111) surfaces and assigned it to a C–O stretching vibration of acrolein adsorbed in a di-σ-C–O configuration.^39^ Furthermore, the COO^–^ asymmetric stretching vibration of a propanoate species on oxide surfaces were found at 1595 cm^–1^ ^38^ and 1565 cm^–1^,^40^ and the C=O stretching frequencies of β-diketone complexes with Cu were reported to appear down to 1524 cm^–1^.^37^ Hence, this IR absorption feature could point to a strong weakening of the carbonyl group because of the tendency of the oxygen to attract electrons C=O ↔ C^+^–O^–^. The polarized form could gain in importance, if the charge is stabilized by the Pd surface. However, a di-σ configuration as observed by Murillo *et al.* for acrolein on Pt–Ni–Pt(111) and Pt–Co–Pt(111) seems unlikely here since a di-σ bounded C–O group would be parallel to the Pd surface and thus not detectable by IRAS due to the MSSR.

In the region below 1500 cm^–1^, IR vibrations are detected at 1468–1455 cm^–1^, 1413 cm^–1^, 1370 cm^–1^, 1358 cm^–1^, 1270 cm^–1^, 1070 cm^–1^, and 990 cm^–1^. IR absorption features at 1468–1455 cm^–1^, 1413 cm^–1^, and 1070 cm^–1^ appear mainly independent of coverage and are assigned to CH_3_ asymmetric bend, CH_2_ bend, and CH_3_ vibrations. The vibrations at 1370 cm^–1^ and 1358 cm^–1^ strongly indicate CH bending or CH_3_ umbrella bending modes; a clear assignment, however, is difficult. Nevertheless, the mode at 1358 cm^–1^ is characteristic of the low-coverage species. With higher certainty, we assigned the peaks at 1270 cm^–1^ and 990 cm^–1^ to CH_2_ twist and CCC asymmetric stretching modes. The latter two vibrational features do not appear in the spectra at higher coverages. Previously, CH_2_ twist vibrations were observed at 1261–1250 cm^–1^, CH_3_ rocking was found at 1098–1093 cm^–1^, and CCC asymmetric stretching modes were reported at 1001–993 cm^–1^.^20^

#### 3.2.4. TPD of propanal

Fig. 2b shows a TPD experiment starting from approximately six layers of propanal on Pd(111). Desorption of acrolein, allyl alcohol, propanal and hydrogen were followed. Neither acrolein nor allyl alcohol are observed in the gas phase. Propanal, however, appears in a strong and sharp desorption peak at 145 K and in a weak feature at 180 K. Hydrogen desorption is observed near 345 K and 470 K. The two hydrogen peaks point to sequential decomposition resulting in a reaction-limited formation of H_2_. The two propanal desorption peaks correspond to desorption of weakly bound (presumably physisorbed) molecules from multilayer at 145 K and stronger bound (chemisorbed) species desorbing form monolayer at 180 K.

### 3.3. Adsorption of allyl alcohol

**Fig. 3:**
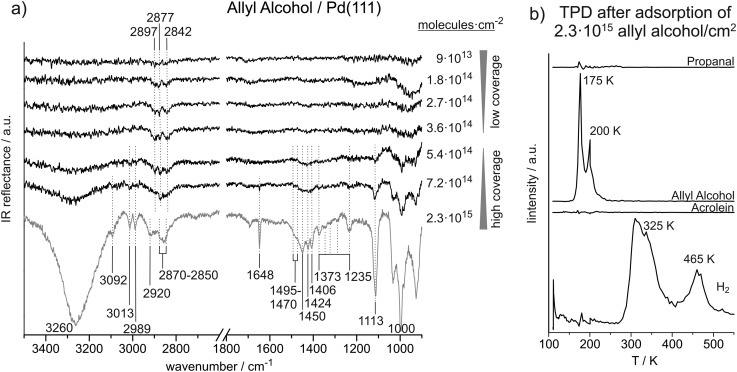
(a) Coverage-dependent IR spectra of allyl alcohol on Pd(111) recorded at 120 K. (b) TPD after deposition of about six layers of allyl alcohol on Pd(111).

Fig. 3a shows a coverage-dependent IRAS study of allyl alcohol adsorbed on Pd(111) at 120 K. We distinguish between a low coverage regime up to 3.6 × 10^14^ ally alcohol molecules per cm^2^ (corresponding to 0.24 allyl alcohol molecules per surface Pd atom) and a high coverage regime starting at 5.4 × 10^14^ molecules per cm^2^ (corresponding to 0.36 allyl alcohol molecules per surface Pd atom). Table 3 summarizes the observed IR vibration frequencies for allyl alcohol at low and at high coverages in comparison to previous studies found in literature.

**Table 3:** Assignment of IR vibrations of allyl alcohol on Pd(111) at 120 K

Mode	IR frequency/cm^–1^		Ref./cm^–1^
	Low coverage	High coverage	
*ν*(O–H···O)		3260	3300^12,43^
*ν*_a_(=CH_2_)		3092	3124–3099,^27^
			3102–3086^26^
*ν*_s_(=CH_2_)		3013	3034–3010^27^
			2996–2992^26^
*ν*(C–H)		2989	3033–3011^27^
			3022^26^
*ν*_a_(CH_2_)	(2897, 2877)	2920	2967–2903^27^
			2948–2934^26^
*ν*_s_(CH_2_)	(2842)	2850–2870	2878–2851^27^
			2880–2854^26^
*ν*(C=C)		1648	1655–1644^27^
			1655^26^
*δ*(CH_2_)		1470–1495	1500–1482^27^
			1463–1453^26^
*δ*(=CH_2_)		1406–1450	1429–1410^26^
			1440–1399^27^
*δ*(=CH_2_)^*a*^		1450	1440^27^
*δ*(=CH_2_)^*b*^		1424	1426^27^
*δ*(=CH_2_)^*c*^ ^,^ ^*d*^		1406	1399, 1409^27^
*ω*(CH_2_)			1399–1317^27^
			1384–1372^26^
*δ*(O–H)		1373–1235	1372–1202,^27^
			1328–1321^26^
*δ*(C–H)			1281–1272^27^
*ν*(C–O)		1113	1111–1033^27^
			1110^26^
*ω*_T_(HC=CH_2_)		1000	1018–841^15^
			1020–950^16^
			1022–1002^17^
			993^13^
			995–985^12^
			1054–988^27^
			1002–995^26^

^*a*^ *gauche–trans*.

^*b*^ *gauche–gauche*.

^*c*^ *cis–trans*.

^*d*^ *cis–gauche*.

#### 3.3.1. Unperturbed allyl alcohol molecule in multilayer

At high allyl alcohol coverages, C–H stretching vibrations are detected at 3260 cm^–1^, 3092 cm^–1^, 3013 cm^–1^, 2989 cm^–1^, 2920 cm^–1^, and in the range of 2870–2850 cm^–1^. The very broad and intense IR absorption near 3260 cm^–1^ is related to O–H stretching in hydrogen bonded OH groups. In previous publications, this vibration was reported near 3300 cm^–1^.^12,43^ The CH_2_ asymmetric and symmetric stretching vibrations are assigned to peaks at 2920 cm^–1^ and 2870–2850 cm^–1^. In previous studies on allyl alcohol in rare gas solutions and in the gas phase, the asymmetric stretching was observed at 2967–2903 cm^–1^ and the symmetric stretching at 2880–2851 cm^–1^.^26,27^ The peak at 3092 cm^–1^ is assigned to the asymmetric stretching of the vinyl-CH_2_ group and the vibrational modes at 3013 cm^–1^, and 2989 cm^–1^ are related to vinyl-CH_2_ symmetric stretching and CH stretching modes. Previously, the vinyl-CH_2_ asymmetric stretching was observed at 3124–3086 cm^–1^.^26,27^ The vinyl-CH_2_ symmetric stretching was found at 3034–2992 cm^–1^ and the CH stretching at 3033–3011 cm^–1^. Hence, the literature values are in good agreement with the vibrational frequencies observed in our study.

The C=C stretching vibration can only be observed at high coverage. It appears at 1648 cm^–1^ and thus 30 cm^–1^ higher as compared to acrolein. In previous studies on allyl alcohol, the C=C stretching vibration was reported at 1655–1644 cm^–1^.^26,27^

In the region below 1500 cm^–1^, IR absorption is found in the ranges of 1470–1495 cm^–1^, 1406–1450 cm^–1^, and 1373–1235 cm^–1^, at 1113 cm^–1^ and 1000 cm^–1^. The features from 1495 cm^–1^ to 1406 cm^–1^ are assigned to CH_2_ scissor vibrations. We relate the higher frequencies at 1470–1495 cm^–1^ to the alkyl-CH_2_ group, while we assign the lower frequencies from 1406–1450 cm^–1^ to the vinyl-CH_2_ group. In literature, scissor deformations of the alkyl-CH_2_ group were found at 1500–1453 cm^–1^ and of the vinyl-CH_2_ group at 1440–1399 cm^–1^.^26,27^ Durig *et al.* predicted theoretically that the vinyl-CH_2_ scissor frequency should strongly depend on the molecular conformation.^27^ According to their results, we tentatively assign the vibration at 1406 cm^–1^ to the *cis–trans* and *cis–gauche* conformer, the absorptions at 1424 cm^–1^ to the *gauche–gauche* and the vibration at 1450 cm^–1^ to the *gauche–trans* species. Multiple IR adsorption features are observed between 1373 cm^–1^ and 1235 cm^–1^, which we assign to CH_2_ wag as well as =C–H and O–H bend vibrations. In previous studies, the exact vibration frequencies were found to depend on the conformation of the molecule.^27^ The pronounced vibration at 1113 cm^–1^ is related to C–O stretching vibration, which was reported in literature between 1033 cm^–1^ and 1111 cm^–1^.^26,27^ The very strong IR absorption near 1000 cm^–1^ is assigned to the CH=CH_2_ *trans*-wag vibration, which is discussed in more detail for acrolein.

#### 3.3.2. Allyl alcohol at low coverage

In the low coverage regime, only a small number of IR absorption features in the C–H stretching region are detected. Weak IR vibration features are observed near 2897 cm^–1^, 2877 cm^–1^, and 2842 cm^–1^. We tentatively assign the two higher wavenumber peaks to CH_2_ asymmetric stretching modes of two different allyl alcohol species and the lower frequency vibration to a CH_2_ symmetric stretching vibration. No vibrations are seen in the frequency range related to C=C stretching or CH_*x*_ deformation vibrations.

#### 3.3.3. TPD of allyl alcohol

Fig. 3b shows the result of a TPD experiment of 2.3 × 10^15^ allyl alcohol molecules cm^–2^ on Pd(111), which corresponds to the highest coverage studied by IRAS. Desorption of allyl alcohol, propanal, acrolein, and hydrogen was followed. Allyl alcohol desorbs in two peaks at 175 K and 200 K, neither propanal nor acrolein desorption were observed. Similar to our studies on propanal and acrolein, a significant amount H_2_ appears in the gas-phase in two peaks at 325 K and near 465 K.

## 4. Discussion

### 4.1. Acrolein on Pd(111)

The coverage-dependent IRAS studies show strong changes in characteristic IR vibration frequencies, especially of the C=O stretching, CH_2_ scissor, and HC=CH_2_ *trans*-wag vibrational modes. These changes point strongly to changes in acrolein adsorption geometry with coverage. Below the coverage of 3.6 × 10^14^ molecules per cm^2^, acrolein forms a sub-monolayer, showing strong deviation in the vibrational spectrum as compared to unperturbed acrolein molecules in a multilayer; the corresponding species are indicated as species A and B in Table 1. Note that the discrimination between A and B is tentative and is rather related to different adsorption geometries than to different chemical nature of the adsorbates. Near the coverage of 3.6 × 10^14^ molecules per cm^2^, which corresponds approximately to accumulation of one acrolein molecule per four Pd surface atoms, acrolein forms a monolayer (species C in the Table 1), which is characterized by considerably smaller changes of the vibrational spectrum as compared to acrolein ice.

At low acrolein coverages (species A and B), weak IR absorption of CH_2_ deformation modes was detected. However, no vibrational bands corresponding to C=O, C=C, or C–C bonds and no IR absorption in the range of C–H stretching vibrations were observed. This distribution of vibrational bands is in sharp contrast to the spectra of unperturbed molecules in acrolein ice (acrolein type D in Table 1). Particularly the absence of the otherwise very intense C=O vibrational band strongly indicates that this band is either strongly perturbed *via* interaction with Pd or cannot be detected by IRAS due to the metal surface selection rule (MSSR).^29–31^ This latter rule allows only vibrations having a component of the dipole moment perpendicular to the metal substrate. Thus, if the C=O bond in acrolein lies flat on the surface at the low acrolein coverages, it would be invisible in IRAS. Both possible reasons – strong perturbation of the C=O bond (*e.g. via* formation of di-sigma complex) as well as the vanishing of the band intensity due to MMSR imply that this bond is parallel to the surface. The same reasons can be put forward in order to explain the missing vibrational bands if the C=C, C–C and C–H stretching regions. It is, however, quite unlikely that all this bands are invisible due to strong perturbation *via* interaction with the Pd substrate, as this scenario would result in very strained surface species. Instead, the vanishing of this band due to flat lying adsorption geometry of acrolein seems to be more feasible in this case. It should be noted that decomposition of acrolein to form CO can be excluded based on our spectra. Acrolein decarbonylation would result in accumulation of CO molecules on the surface, which have a very large dipole moment and should be clearly visible.

It should be noted that MMSR is not a universal rule as in strongly interacting systems even the bands lying parallel to the surface might become visible in IRAS due to surface electron density being “pumped” in and out of the metal surface thus producing a strong dynamic dipole moment perpendicular to the surface. However, this scenario does not apply in our case to C=C and C=O bonds of acrolein, as these vibrations are not seen at low coverage and therefore the electron density pumping into the parallel lying bands can be safely excluded.

At increasing acrolein coverages, changes in the CH_2_ scissor vibration frequency were detected. While it was significantly shifted with respect to acrolein ice by 25 cm^–1^ in the lowest coverage limit (species A, 1400 cm^–1^), the frequency changes to the value of 1425 cm^–1^ characteristic for an unperturbed molecules. We tentatively refer these changes to the formation of the species B. The different CH_2_ scissor vibration frequencies of species A and B are rather related to a different strength and/or degree of perturbation of the corresponding group upon interaction with Pd. The unperturbed vibration of the CH_2_ group in species B points to a weaker interaction of this group with the surface, which might arise *e.g.* from a more upright orientation of this group and, consequently, larger distance between the CH_2_-entity and Pd surface. Interestingly, the C=O band remains still invisible in IRAS of these species suggesting that the CH_2_ groups tilts at lower coverages than the C=O group. A very similar scenario was observed for the coverage-dependent changes of a similar α,β-unsaturated ketone isophorone, whose changes in the adsorption geometry were followed by combination of IRAS and NEXAFS.^41,44^ For this molecule, we also found that the initial flat adsorption geometry changes to an upright geometry with increasing coverage, but the C=C bond acquires the tilted geometry at lower coverages than the C=O bond, which is accompanied by a strong perturbation of both geometrical and electronic structure of this molecule.

It should be noted that the IR absorption features of the sub-monolayer species are very weak and a clear identification of the molecular structure of the adsorbates is hardly possible. Hence, the most reliable information obtained by the IR studies at sub-monolayer coverages may rather be related to the adsorbates' geometry than to their exact molecular structure. The absence of the C=O, C=C, and C–C bond stretching vibration signals reveals a flat-lying molecular plane on the Pd(111) surface.

At the exposure 3.6 × 10^14^ acrolein molecules per cm^2^, a clearly detectable C=O stretching vibration appears in the spectrum at 1663 cm^–1^. This observation indicates that the C=O bond acquires more upright configuration with respect to the surface plane. The frequency is shifted by 17 cm^–1^ as compared to the unperturbed molecule in acrolein ice, pointing to a weakening of the C=O bond due to a strong interaction with the metal surface. Note that species C related to the monolayer coverage can be only observed in a very narrow coverage range close to one acrolein molecule per four Pd surface atoms. TPD spectra obtained for the monolayer coverage show complete decomposition of the entire acrolein overlayer indicating that the whole layer is strongly interacting with the surface. Previous DFT calculations show that acrolein may adsorb on a Pd(111) surface in an η_4_ mode, in which it would block four Pd atoms.^2^ This theoretical prediction is in good agreement with our experimental data suggesting that adsorption of one acrolein molecule per four Pd surface atoms results in formation of a homogeneously distributed and strongly bonded monolayer of flat-lying molecules.

Finally, at the highest investigated coverages, acrolein adsorbs in a multilayer (species D). The corresponding IR vibrational bands appear at frequencies similar to that reported in literature for gas-phase acrolein. Therefore, the IR spectrum of species D provides a good reference for vibrations of mainly unperturbed adsorbed molecules.

### 4.2. Propanal on Pd(111)

The vibration frequencies of propanal exhibit a very strong coverage dependence, which gives some insight into the adsorbate structures formed on Pd(111). Similar to acrolein, we observe the transition from the sub-monolayer to multilayer adsorption regime in the range from 3.6 × 10^14^ to 5.4 × 10^14^ molecules per cm^2^. It is particularly interesting to compare the vibrational signatures of adsorbed acrolein and propanal species as they differ only in the presence of the conjugated π system in acrolein.

Already at sub-monolayer coverages, propanal exhibits a number of IR vibrational modes. A variety of CH_3_, CH_2_, CH, C–C–C, as well as C=O vibration have been identified at low propanal coverages. While all vibrational modes of the CH_2_ group appear at identical frequencies as for unperturbed molecules at multilayer coverages, the CH_3_ stretching vibrations are significantly affected by the Pd surface at sub-monolayer coverages. CH_3_ asymmetric and symmetric stretching vibrations have been observed at 2960 cm^–1^ and 2876 cm^–1^ and thus 27 cm^–1^ and 8 cm^–1^ lower than in the unperturbed species relative to the multilayer. The CH_3_ symmetric bending and CH_3_ rocking vibrations appear near 1360 cm^–1^ and 1070 cm^–1^, respectively, which is approximately 30 cm^–1^ lower than in multilayer species. However, the most drastic changes have been observed for the stretching vibration frequency of the C=O group as a function of increasing coverage.

On the one hand, the pronounced coverage-dependent changes of the C=O stretching vibration frequency can be indicative of a strong interaction of this group with the metal surface. In particular, close to the monolayer coverage, three vibrational bands on the C=O vibrational were observed: at 1663 cm^–1^, at 1695 cm^–1^ and at 1728 cm^–1^. While the first band at 1663 cm^–1^ appears and quickly saturates in the sub-monolayer regime, the second band at 1695 cm^–1^ grows in intensity up the coverage of *ca.* 5.4 × 10^14^ propanal molecules per cm^2^. The third band at 1728 cm^–1^ continuously grows in intensity with increasing coverage and represents one of the major vibrational features in propanal ice. The vibrational band at 1663 cm^–1^ is most likely related to surface species formed at sub-monolayer coverages or close to monolayer and points to a major weakening of the C=O bond in coverage range due to strong interaction of propanal with Pd(111) and/or strong perturbation of its electronic structure. High intensity of the C=O bond near the full monolayer coverage indicates a rather upright orientation of this bond on Pd(111). This observation is in contrast to acrolein, which was found to adsorb in a flat-lying adsorption geometry with both C=C and C=O binds being nearly parallel to the surface even at the smallest investigated coverages. The second C=O vibrational band (1695 cm^–1^) is rather indicative of formation of the second propanal layer, which is less perturbed by the interaction with Pd and therefore exhibits the C=O vibrational frequency lying very closer to the gas phase and ice value (1728 cm^–1^). The appearance of the vibrational band at 1728 at 5.4 × 10^14^ molecules per cm^2^ indicates the onset of propanal ice formation. As the frequency does not change in the next layers, it can be safely assumed that a noticeable perturbation of the electronic structure of propanal is possible only for molecules in the first two adsorption layers.

Significant differences between acrolein and propanal were observed in the region of the C–H stretching vibrations. While these vibrations cannot be seen at sub-monolayer coverages of acrolein, these modes are clearly visible already at the lowest coverage of propanal. This observation indicates a different ordering of the CH_*x*_ groups of propanal and acrolein on the Pd surface. In acrolein, the dynamic dipole moment of these vibrations must be close to parallel to the metal surface in order to become invisible in IRAS due to MSSR, while the same vibrations in propanal obviously exhibit rather large perpendicular component of the dipole moment. This observation is consistent with the more upright adsorption geometry of propanal discussed above, resulting in the C=O vibrational peaks of high intensity observed already at close to monolayer coverages.

It is important to note that in the high coverage regime the C–H stretching vibrations of propanal appear at significantly higher wavenumbers compared to acrolein (2752–2987 cm^–1^ for propanal and 2705–2857 cm^–1^ for acrolein). These differences result most likely from the different chemical environment of the CH_*x*_ species in acrolein *vs.* propanal, *i.e.* from the presence or absence of the conjugated π-system of double bonds. For instance, vibrations of the aldehyde-C–H groups in acrolein gives rise to peaks at 2705 cm^–1^ and 2766 cm^–1^, while the same modes in propanal are observed at 2752 cm^–1^ and 2868 cm^–1^. The CH_2_ stretching vibrations in propanal appear at 2943 cm^–1^ and 2904 cm^–1^ and thus at significantly higher wavenumbers as the =CH_2_ vibration in acrolein at 2857 cm^–1^ and 2820 cm^–1^, which involve a C atom at an unsaturated C=C bond.

It should be pointed out that the differences in the intensities as well as in the frequencies of the C–H stretching vibrations can allow an independent *in situ* monitoring of adsorbed acrolein and its hydrogenation product propanal under the reaction conditions of acrolein hydrogenation.

The different propanal surface species formed on Pd in the coverage rage 3.6 × 10^14^ to 5.4 × 10^14^ molecules per cm^2^, can be clearly correlated to the temperature-programmed desorption experiments. TPD experiments of propanal starting from the multilayer coverage show desorption of intact propanal in multilayer at 145 K and monolayer at 180 K. Hydrogen desorption peaks are observed at 345 K and 470 K. This desorption pattern indicates that not the entire overlayer of propanal decomposes to produce hydrogen and some fraction of propanal can desorb as an intact molecule. The species with the lower desorption temperature of 145 K is related to the weakly bound propanal characterized by the mostly unperturbed C=O vibration at 1728 cm^–1^. The propanal species desorbing at 180 K is most likely related to the propanal adsorbed in the second layer and characterized by the C=O vibrational frequency of 1693 cm^–1^ maybe with some fraction of propanal adsorbed in the first monolayer with the C=O stretching frequency of 1663 cm^–1^. It is also very likely that the sub-monolayer type of species (C=O stretching frequency of 1663 cm^–1^) undergo partial decomposition in a TPD run resulting in hydrogen desorption.

### 4.3. Allyl alcohol on Pd(111)

At low coverages (up to 3.6 × 10^14^ molecules per cm^2^) of allyl alcohol on Pd(111), only weak IR absorption features related to C–H stretching vibrations were detected. The absence of the otherwise strong vibrational bands in the range of C–O and O–H frequencies (near 1110 cm^–1^ and 3260 cm^–1^, respectively) point to a flat lying adsorption geometry of allyl alcohol at sub-monolayer coverages. This adsorption geometry is additionally corroborated by the fact that some low intensity bands in the range of the CH_2_ stretching vibrations (2840–2900 cm^–1^) can be observed even at sub-monolayer coverages. These bands have small dipole moments, which normally results in the low intensity of these bands as compared to the C–O and O–H vibrations possessing large dipole moments. The fact that these low intensity bands are present and their frequency correspond well to the frequencies in allyl alcohol ice mean that the molecules are adsorbed on the surface and are most likely intact; however, their C–O and O–H vibrational bands are not visible due to flat lying adsorption geometry.

At allyl alcohol coverages higher than 3.6 × 10^14^ molecules per cm^2^, a transition from sub-monolayer coverage to a mono- and multilayer coverages is observed. This transitions is characterized by evolution of a large number of bands characteristic for the O–H, CH_*x*_, C–O, and C=C groups at the frequencies nearly identical to those reported in previous studies on unperturbed allyl alcohol molecules in the gas phase and rare gas solutions. Appearance of these vibrational bands clearly indicates the change of the adsorption geometry from the flat lying to a more tilted geometry at higher coverages. Finally, these vibrational bands merely grow in intensity without undergoing any significant shifts, which indicates the growth of an allyl alcohol multilayer. In the multilayer regime, all recorded vibrational frequencies are in good agreement with the literature values reported earlier. This observation suggests a transition from the monolayer to multilayer regime in the range of 3.6 × 10^14^ to 5.4 × 10^14^ allyl alcohol molecules per cm^2^ and thus approximately at the same surface coverage as determined for acrolein and propanal.

TPD studies on multilayers of allyl alcohol on Pd(111) show desorption of intact allyl alcohol at 175 K and 200 K and hydrogen at 325 K and 465 K. Desorption of allyl alcohol at 175 K is clearly related to multilayer regime, while the peak at 200 K can be assigned to desorption from the monolayer. It is interesting to note that a fraction of the allyl alcohol might desorb intact from the monolayer, while the rest of the monolayer successively decomposes resulting in hydrogen desorption peaks at 325 K and 465 K. Interestingly, the high-temperature H_2_ desorption in propanal and allyl alcohol TPD experiments appears approximately at the same temperature. This observation indicates that the hydrocarbon fragments remaining on the surface up to 470 K might be identical for both molecules propanal and allyl alcohol. This temperature range was previously reported to be characteristic of decomposition of the CH_*x*_ groups.^35^ The low temperature H_2_ peak observed in allyl alcohol decomposition, is by about 20 K lower than that one recorded for propanal. This difference is most likely related to the lower stability of the hydrogen atoms attached to the C=C bond in allyl alcohol. In agreement with this hypothesis, the low-temperature H_2_ peak observed in acrolein decomposition (the onset at 315 K) lies much closer to the corresponding peak of allyl alcohol (320 K) than of propanal (340 K). It might be concluded from these observations that the molecules containing C=C bonds are more prone to decompose as compared to their saturated counterparts.

The desorption of allyl alcohol from Pd(111) occurs at 175 K and 200 K, compared to the desorption temperature of propanal at 145 K and 180 K. Redhead analysis of the desorption curves^45^ results in the estimated binding energies of 50 kJ mol^–1^ and 44 kJ mol^–1^ for the mono- and multi-layer allyl alcohol species and 45 kJ mol^–1^ and 36 kJ mol^–1^ for the two propanal species. This difference in the binding energies of two potential products of acrolein hydrogenation might play an important role for the overall hydrogenation process as propanal is expected to desorb faster than allyl alcohol at a given temperature.

It is interesting to note that in sub-monolayer coverage regime acrolein and allyl alcohol adopt a flat-lying adsorption geometry on the Pd(111) surface, while propanal is strongly inclined with respect to Pd(111) surface plane, interacting with the surface primarily through the C=O group. This result is in good agreement with our previous study on the adsorption geometries of an unsaturated α,β-ketone isophorone and its saturated counterpart – trimethylcyclohexanone. By using a combination of IRAS and NEXAFS, we were able to show that isophorone adopts a flat-lying adsorption geometry on Pd(111) at sub-monolayer coverages, while TMCH is attached to the surface *via* the C=O bond and is strongly tilted.^41^ It might be speculated that the presence of the C=C bond plays an important role in determining the adsorption geometry of surface species and, as a result, could explain the selectivity of C=C *vs.* C=O bond hydrogenation in α,β-unsaturated aldehydes. Further theoretical calculations are needed in order to understand this issue. The strong changes of the adsorbates structure from the unsaturated to the saturated aldehyde is expected to play a crucial role in explaining the selectivity of C=C *vs.* C=O bond hydrogenation in α,β-unsaturated aldehydes.

## 5. Conclusions

Adsorption of acrolein, propanal and allyl alcohol on Pd(111) was investigated in this study by a combination of infrared reflection absorption spectroscopy and temperature programmed desorption. The evolution of IR vibrational bands measured as a detailed function of adsorbates coverage in the broad range starting from sub-monolayer up to multilayer coverages provides deep insights into the adsorbate structures formed on Pd(111). For all three compounds, we have found mostly unperturbed molecular structures in the multilayer regime and observed strong perturbation of the molecules upon interaction with Pd. A transition from the sub-monolayer to the multilayer regime has been observed in the range from 3.6 × 10^14^ to 5.4 × 10^14^ molecules per cm^2^ for all three compounds.

For adsorption of acrolein in the sub-monolayer regime, two types of surface species (A and B in Table 1) were spectroscopically identified that adsorb with C=O, C=C and C–C bonds parallel to the metal surface. Remarkably, only deformation vibrations involving the =CH_2_ group have been observed at the lowest acrolein coverages. In contrast, at a coverage close to a monolayer (give the number of molecules) a more upright orientation of the C=O bond was observed. The corresponding vibrational frequency was found to be strongly red-shifted from the gas phase and acrolein ice value, indicating a significant weakening of the C=O bond in acrolein by the interaction with Pd and strong distortion of the conjugated π system. At higher coverages, the mainly unperturbed acrolein species is formed with the vibrational frequencies in good agreement with the previously reported values for gas phase acrolein.

Three coverage regimes were identified in the IRAS experiments on propanal adsorption, which can be tentatively assigned to formation of the first and second propanal layer as well as building up the propanal multilayer. In the sub-/monolayer regime, the C=O vibrational band (1663 cm^–1^) was observed to strongly interact with Pd, resulting in a strong shift to lower wavenumbers as compared to unperturbed molecules. High intensity of this band as well as C–H stretching vibrations points to a strong inclination of adsorbed propanal molecules with respect to the Pd surface even at sub-monolayer coverages. Close to the monolayer coverage (give the number of molecules), the onset of a second C=O vibrational band at higher frequencies (1693 cm^–1^) is observed that grows in intensity with increasing coverage and later saturates. We interpret this observation as formation of a second propanal layer characterized by lower binding to the underlying metal substrate and weaker perturbation of the electronic structure. At higher coverages, a multilayer of propanal was formed characterized by essentially non-perturbed IR spectra compared to gas phase propanal.

In the case of allyl alcohol, identification of adsorbates at low coverages is difficult due to weak IR absorption and most likely flat-lying adsorption geometry of allyl alcohol. We were able to identify a few C–H vibration features in the low-coverage regime. With increasing coverage, however, a transition to the multilayer regime was detected and a large number of distinct IR vibrational modes were observed with the vibrational frequencies characteristic for unperturbed gas phase molecules.

We found pronounced differences in the adsorbates' structures of acrolein, propanal and allyl alcohol on the Pd(111) surface. While at sub-monolayer coverages acrolein and allyl alcohol species adsorb with the molecular chain parallel to the surface, propanal adsorbs in a strongly tilted geometry with respect to Pd(111) surface plane, interacting with the surface primarily through the C=O group. This finding might suggest that the presence of the C=C bond plays an important role in determining the adsorption geometry of surface species. The strong changes of the adsorbates structure from the unsaturated to the saturated aldehyde is expected to play a crucial role in explaining the selectivity of C=C *vs.* C=O bond hydrogenation in α,β-unsaturated aldehydes.
